# Bulbar Onset Amyotrophic Lateral Sclerosis: A Case Report

**DOI:** 10.31729/jnma.8098

**Published:** 2023-03-31

**Authors:** Kabindra Rai, Nilshan Rai, Milan Kumar Chhantel Thapa, Rajan Shrestha, Sampurna Singh, Monika Thapa

**Affiliations:** 1 KIST Medical College and Teaching Hospital, Mahalaxmi, Lalitpur, Nepal; 2 Department of Neurology, National Neuro Center, Maharajgunj, Kathmandu, Nepal

**Keywords:** *amyotrophic lateral sclerosis*, *aspiration pneumonia*, *case reports*, *edaravone*

## Abstract

Amyotrophic lateral sclerosis is a rare, progressive, incurable neurodegenerative disorder that affects motor neurons leading to progressive muscle weakness, disability, and eventually death. A 45-year-old male, initially presented with hoarseness, flickering of tongue, and intermittent aspirations. In course of three years, patient developed motor aphasia, frequent aspirations and an inability to hold his neck. Patient was diagnosed with a bulbar onset type of amyotrophic lateral sclerosis on the basis of neurodegenerative features with normal radiographic imaging. For the prevention of recurrent aspiration pneumonia, he was managed with a percutaneous endoscopic gastrostomy tube. As he started developing respiratory failure tracheostomy was performed and kept on a continuous bi-level positive airway pressure ventilator, in the meantime, two courses of injection Edaravone were given. Early evaluation, diagnosis and management of the condition is a cornerstone for better prognosis of disease and survival.

## INTRODUCTION

Amyotrophic lateral sclerosis (ALS) is a progressive neurodegenerative disorder mainly affecting motor neurons in the brain and spinal cord.^1,2^ Different presentations of ALS are limb onset ALS, bulbar onset ALS, primary lateral sclerosis, and progressive muscular atrophy.^3^ Bulbar onset ALS presents most commonly with dysarthria, tongue wasting, tongue fasciculation, dysphonia, and later dysphagia. The worldwide ALS incidence is approximately 1.59 per 100,000 persons per year with a mean survival duration of two to five years of disease onset.^4^ A 45-year-old male present with acute onset of shortness of breath and multiple episodes of vomiting.

## CASE REPORT

A 45-year-old male came to our emergency department with chief complaints of acute onset shortness of breath and multiple episodes of vomiting for one day. Dry sensation in the throat, a flickering of tongue, and hoarseness had started 3 years back, for which he had visited the ear, nose and throat outpatient department, a diagnostic nasopharyngeal-laryngoscopy was performed and had shown an adduction gap of the vocal cord.

He gradually began to develop frequent aspirations while eating and drinking, hoarseness worsened into aphasia and could not hold his head erect. He had to be admitted and managed for recurrent aspiration pneumonitis multiple times. At first, he was managed with a nasogastric feeding tube to prevent aspiration later he had undergone percutaneous endoscopic gastrostomy 4 months back before the presentation for feeding.

At presentation, examination showed dysarthria in which speech comprehension was present, phonation was present but articulation was absent with exaggerated jaw jerk, absent gag reflex, inability to protrude tongue rather than fasciculation in an effort, power of neck and shoulder muscles were diminished, other neurological examinations were normal. He had normal bowel and bladder habits. There was no significant personal or family history. There were not any lesions in the brain and spinal cord in the magnetic resonance imaging (MRI) brain and angiography. But as 9^th^, 10^th^, 11^th^ and 12^th^ cranial nerves were involved with normal magnetic resonance imaging and angiography findings, based on the clinical evaluation done by a neurologist and imaging findings, a diagnosis of bulbar onset type of amyotrophic lateral sclerosis was made.

He was admitted for management of aspiration pneumonitis secondary to bulbar onset ALS with hyponatremia with hypokalemia with hypocalcemia. After a few hours of admission to the intensive care unit (ICU), a sudden drop in his oxygen saturation upto 60% along with weak respiratory effort indicated the need for intubation and ventilation. His respiratory efforts did not improve until the seventh day on the ventilator so an elective tracheostomy was performed for long-term ventilation. In the meantime, intravenous injection edaravone therapy was given for two courses of 10 days regimen with 30 mg twice a day infusion with 10 days intervals in between the course. This significantly showed improvement in breathing effort so with portable Bi-level positive airway pressure (BiPAP) mode of ventilation, he was shifted to the general ward on the 19^th^ day. With correction in other medical conditions, he was discharged on the 30^th^ day of admission with a percutaneous endoscopic gastrostomy tube and tracheostomy tube with a portable BiPAP ventilator in situ with the advice of lifestyle adjustments and regular follow-up.

## DISCUSSION

ALS is a rare progressive neurodegenerative disorder mainly affecting motor neurons and may cause muscular paralysis. The symptoms can be of spinal onset such as stumbling, weakness of arms and legs or bulbar onset as slurred or hypernasal speech, dysphagia, hoarseness, and facial weakness,^1^ In our case, the patient had the main symptoms of the bulbar onset ALS, such as slurring of speech, hoarseness, swallowing difficulty and inability to hold the head.

Globally, the mean age of onset of symptoms varies from 58-63 years for sporadic ALS and 40-60 years for familial ALS.^3,4^ In our case the age of the patient is 45 years which was comparable to other similar cases. Men have a higher risk of developing sporadic limb onset ALS globally but in our case, the patient was of bulbar onset type of ALS.^1^

Respiratory failure and nutritional deficiencies are significant concerns for patients with ALS which is similar to this case presenting with aspiration pneumonitis.^1^ The mean survival age is 2-5 years for patients with ALS but in this case, the patient's survival period exceeds.^1^ The main risk factors are a combination of genetic and environmental factors such as smoking, physical exercises, head injury, exposure to pesticides, and viral infection however the exact relationship between risk factors and disease is yet to be established which was similar to this case. There are more than 20 gene mutations found that are known to cause ALS but in this case we did not do any gene analysis.^1^ The major gene is the C9ORF72 gene, responsible for 30% to 50% of familial ALS and 7% of sporadic ALS.^1^

In many different studies, the diagnosis of ALS is mainly done by clinically, laboratories and imaging tests are used to differentiate from other ALS-mimicking diseases but in our case diagnosis of ALS was done by clinical methods, laboratories and imaging method involving 9^th^, 10^th^, 11^th^ and 12^th^ cranial nerves that caused hoarseness, dysarthria, flickering of the tongue, dysphagia and recurrent aspirations without any family history of similar illness, reduced mouth closing, chewing problem, brisk gag and jaw reflex which similar to this study.^2^

Delayed diagnosis is one of the major limitations in our case for intervening in the progression of the disease. Despite presenting 18 months earlier at the ear, nose and throat outpatient department with mentioned symptoms definitive diagnosis was made lately. Other investigations like nerve conduction tests, electromyography, trans-cranial magnetic stimulations and other laboratory or genetic testing were not done due to the inaccessibility of the facility.

Riluzole and edaravone are currently recommended food drug administration-approved medications to prolong the survival of patients significantly improving the Revised Amyotrophic Lateral Sclerosis Functional Rating Scale (ALSFRS-R) score and pulmonary function tests. Other drugs like albrioza, NU-9, tofersen and arimoclomol are under different phases of clinical trials.^5-7^ In our case edaravone showed significant improvement in patients' condition.

Although the incidence of ALS is very low, a clinician should always keep ALS as a differential in mind for patients presenting with neurological complaints like hoarseness, dysphagia, fasciculation, motor weakness or stiffness so that early workup, diagnosis and intervention can be possible. Early intervention may prolong the survival age and progression of the disease.

In conclusion, early evaluation, diagnosis and management of the condition is a cornerstone for better prognosis of disease and survival. Thorough counselling of the condition, intervention and prognosis to the patient and the patient party is much needed for preparedness for financial and psychosocial challenges and compliance with the treatment and follow-ups.
